# Participating in a fruit and vegetable intervention trial improves longer term fruit and vegetable consumption and barriers to fruit and vegetable consumption: a follow-up of the ADIT study

**DOI:** 10.1186/s12966-015-0311-4

**Published:** 2015-12-18

**Authors:** Charlotte E Neville, Michelle C McKinley, Claire R Draffin, Nicola E Gallagher, Katherine M Appleton, Ian S Young, J David Edgar, Jayne V Woodside

**Affiliations:** 1Centre of Excellence for Public Health, Queen’s University Belfast, School of Medicine, Dentistry and Biomedical Sciences, Institute of Clinical Science B, Grosvenor Road, Belfast, BT12 6BJ UK; Department of Psychology, Bournemouth University, Dorset, UK; Regional Immunology Service, Kelvin Building, Royal Victoria Hospital, Grosvenor Road, Belfast, BT12 6BN UK

**Keywords:** Randomized controlled trial, Fruit, Vegetables, Barriers, Adherence

## Abstract

**Background:**

Fruit and vegetable (FV) based intervention studies can be effective in increasing short term FV consumption. However, the longer term efficacy of such interventions is still unclear. The aim of the current study was to examine the maintenance of change in FV consumption 18-months after cessation of a FV intervention and to examine the effect of participating in a FV intervention on barriers to FV consumption.

**Methods:**

A follow-up of a randomised controlled FV trial in 83 older adults (habitually consuming ≤2 portions/day) was conducted. At baseline, participants were assigned to continue consuming ≤2 portions FV/day or consume ≥5 portions FV/day for 16-weeks. We assessed FV intake and barriers to FV consumption at baseline, end of intervention and 18-months post-intervention.

**Results:**

At 18-months, mean FV intakes in both groups were greater than baseline. The 5 portions/day group continued to show greater increases in FV consumption at 18-months than the 2 portions/day group (*p* < 0.01). At 18-months, both groups reported greater liking (*p* < 0.01) and ease in consuming FV (*p* = 0.001) while difficulties with consuming FV decreased (*p* < 0.001). The 2 portions/day group reported greater awareness of FV recommendations at 18-months (*p* < 0.001).

**Conclusions:**

Participating in a FV intervention can lead to longer-term positive changes in FV consumption regardless of original group allocation.

**Trial registration:**

Clinical Trials.gov NCT00858728.

## Background

Increasing fruit and vegetable (FV) consumption is widely accepted as being an important component of a healthy lifestyle. The importance of FV for the prevention of chronic conditions, including cardiovascular disease, certain cancers and diabetes, has resulted in global public health recommendations to consume at least 400 g/day or a minimum of five servings of FV/day [1]. However, despite the evidence that FV are, overall, beneficial to health, FV intakes throughout Western populations remain low [2, 3]. More specifically, a USDA report highlighted that less than 50 % of older adults eat the recommended five servings of FV/day [4].

Finding methods to encourage older adults to increase their FV consumption and sustain this behaviour in the long-term remains a challenge. Short-term increases in FV intake have been achieved in a number of studies that have used intensive strategies, such as intensive dietetic support or food provision, to achieve increased FV consumption thus allowing examination of the effects of FV on risk factors and biomarkers of disease [5]. In a recent randomised controlled trial, we demonstrated that taking part in a FV intervention was effective in increasing FV intake after 16-weeks [6–8]. What is unknown is whether taking part in such studies, either as an intervention or control participant, has a lasting effect on FV intake. It is plausible that such interventions may have longer-term effects on dietary behavior as some of the well-established barriers to FV intake will be addressed by the compliance strategies used in these efficacy interventions. While some studies have examined the effect of a FV intervention on FV consumption the majority of these studies had short duration of follow-up (3 months–12 months) [9–12], were conducted in younger age groups [11, 13], and tended to focus on individual barriers to FV consumption rather than considering a range of barriers that may affect FV consumption.

Barriers such as poor nutritional knowledge, cost, dislike of FV, lack of awareness of FV recommendations and practical issues have all been found to be highly predictive of low FV intakes [14–18]. In older people, poor nutritional knowledge and practical issues related to frailty and age-related dysfunction may contribute more substantially to low FV intakes than other barriers [15, 19].

The current FV intervention study addressed several of the above barriers (knowledge, cost, access, practical issues) in order to achieve the desired increase in FV intake. The aim of the follow-up phase of this study, conducted 18-months after the intervention ceased, was to examine if the intervention had a lasting effect on FV intake, whether the extent of longer-term change varied according to original group allocation (i.e. intervention or control) and whether participating in the intervention had an immediate or longer-term effect on perception of barriers to FV consumption.

## Methods

This study (named the Ageing and Dietary Intervention Trial (ADIT)) was a randomised controlled parallel group trial, primarily designed to examine the effect of 5 portions FV/day compared to ≤2 portions/day on clinically relevant immune function markers. Details regarding the intervention design, inclusion/exclusion criteria and the primary and secondary endpoints have been published elsewhere [6–8]. In brief, 83 healthy, free-living males and females, aged ≥65y with a habitual FV consumption of ≤2 portions/day participated. Exclusion criteria included those on special diets, taking nutritional supplements or medications known to affect immune status or nutrient absorption; excessive alcohol consumption (>28 units/week men, >21 units/week women); BMI > 35 kg/m^2^; history of diabetes or dementia; Pneumovax II vaccination within previous 2-y; inability to provide informed consent; any other problem which would prevent adherence to a high FV diet; or recent infection (< 3-weeks since completion of any antibiotic course or symptoms of viral illness). Participants were recruited through press releases to local media, older people’s networks, newsletters and bulletins and also through contact with older peoples’ community groups and hospital outpatient clinics. Eligible individuals gave written informed consent prior to participation. All subsequent study visits and data collection took place in the participants’ own homes. The study was approved by the Office for Research Ethics Committees Northern Ireland and eligible individuals gave written informed consent prior to participation.

Participants were randomised to either consume ≥5 portions of FV/day (intervention group), or to follow their normal diet (≤2 portions FV/day; control group) for 16-weeks. FV were delivered weekly to all participants, free of charge. During the intervention, participants in the 5 portions/day group received personal dietary advice and education (written and verbal) regarding practical ways of incorporating FV into their diet and experimenting with new types of FV they may not have previously eaten. Compliance was also encouraged by providing the participants in the 5 portions/day group with recipe ideas and daily meal suggestions. The advice given was tailored towards the physical capabilities of the participant and without compromising their energy intake. As a means of supporting dietary compliance, participants (regardless of group allocation) also received a weekly home delivery of FV, free of charge. The type and amount of FV provided to each participant in the FV delivery was based on individual FV preferences and on group allocation, respectively. Participants were given a list of a wide range of FV from which they could select their FV. Each participant was encouraged to try a broad range of FV throughout the 16-weeks. At the end of the intervention period, participants in the 2 portions/day group received the same dietary advice as the 5 portions/day group regarding ways to increase their FV intake.

Habitual FV intake was assessed at baseline and at the end of the intervention as part of a 7-day diet history interview. Food Portion Sizes were used as an aid in quantifying intakes [20]. Dietary data were analysed using WISP v3.0 (Tinuviel Software, Warrington).

All participants completed a questionnaire at baseline and at the end of the intervention to assess barriers to FV consumption. This questionnaire, based on a FV barriers questionnaire previously developed by Appleton et al. [18], contained twenty-one closed statements focusing on various aspects of FV consumption. Participants were asked to strongly agree, agree, neither agree nor disagree, disagree or strongly disagree, responses were scored as 2, 1, 0, −1, −2 respectively. At the end of the questionnaire participants were also asked whether or not they perceived themselves to be eating enough FV. Full details regarding the content of the barriers to FV consumption questionnaire have been published previously [18].

All participants who participated in the 16-week trial were telephoned 18-months post-intervention and invited to participate in a telephone interview, the purpose of which was to assess participants’ current dietary intake, including FV, and to assess changes in barriers to FV consumption. The questionnaires and methods employed at the 18-month assessment were the same as those used at baseline and at the end of the intervention.

### Statistical analysis

Data were analysed using SPSS version 20.0 (SPSS Inc., Chicago, US). Descriptive statistics were obtained for each variable of interest according to the FV allocation group. Normally distributed continuous variables were summarised using mean ± SD.

The responses to the barriers to FV questionnaire were grouped into the following factors: ‘willingness to change’, ‘liking’, ‘ease’, ‘difficulties’ and ‘awareness’, as determined during questionnaire development. For each factor, a barriers score was calculated per individual, whereby the responses to questions contributing to each factor were summed and divided by the number of questions for that factor. This resulted in a score (for each factor) of 2 to −2 for each individual [18].

Data were analysed using repeated measures ANCOVA, to investigate differences over time (0–16 weeks, and 0–18 months) between groups. Between-group comparisons of baseline values were made using independent samples t-tests and chi-square analysis for continuous and categorical variables respectively. Initial analyses of between-group differences in participant variables revealed differences between groups in gender (2 portions/day = 18 % male; 5 portions/day = 49 % male), and gender is a known predictor of FV intake [3], thus gender was used as a covariate in all analyses. Within-group comparisons of change in outcome variables were made using paired samples *t*-test. The significance level for all tests was *p* < 0.05. Linear regression analyses were also conducted to examine the relationship between FV consumption at the end of the intervention and at 18-months post-intervention and barrier scores. FV consumption was predicted by change scores for the five barriers, FV consumption at baseline, intervention group, and gender. Change in FV consumption was also investigated using change scores for the five barriers, intervention group and gender. Data are only presented for those who completed baseline, end of intervention and 18-months post-intervention assessments.

## Results

Baseline physical characteristics of participants according to their allocated FV group have previously been reported [6–8]. In brief, 83 participants completed baseline assessments. Of these 83 participants, 41 were randomised to the 2 portions/day group and 42 to the 5 portions/day group. Eighty-two participants (99 %) completed the 16-week intervention while 80 (96 %) participated in the 18-month post-intervention assessment. Of those who participated in the post-intervention assessment, 39 had originally been randomised to the 2 portions/day group while 41 had been randomised to the 5 portions/day group.

Similarly to what was previously reported [6–8], participants completing the 18-month post-intervention had a mean baseline age of 71.1 y (SD 4.8) and were primarily female. More men were included in the 5 portions/day group than in the 2 portions/day group. Participants in the 5 portions/day group were significantly taller and heavier than those in the 2 portions/day group although their BMI did not differ significantly. Significantly more women in the 2 portions/day group had previously used hormone replacement therapy (HRT) compared to those in the 5 portions/day group. No other significant baseline differences were evident between the two FV groups.

The change in fruit consumption, vegetable consumption and total FV consumption between baseline and the end of the intervention and between baseline and 18-months post-intervention is presented in Table 1. In general, participants reported consuming more fruit than vegetables. As previously reported [6–8], there was a significant between-group difference in the change in fruit, vegetable and FV consumption between baseline and the end of the intervention, with greater increases in the 5 portions/day group compared to the 2 portions/day group (smallest F(1,79) = 53.64, *p* < 0.001). After 18-months, mean FV intakes in both groups were greater than at baseline, albeit approaching significance (F(1,77) = 3.63, *p* = 0.06), and in total FV intakes, fruit intakes and vegetable intakes increases remained greater in the 5 portions/day group compared to the 2 portions/day group (smallest F(1,77) = 6.16, *p* = 0.01). Within-group differences showed that in the 5 portions/day group, fruit intake, vegetable intake and total FV consumption increased significantly between baseline and follow-up (smallest t(40) = 7.72, *p* < 0.01), while lesser effects were found in the 2 portions/day group (largest t(38) = 4.64, *p* < 0.01). Overall, at 18-months, 40 participants (98 %) in the 5 portions/day group reported an increase in FV consumption while one (2 %) reported a decrease in FV consumption, relative to baseline. In the 2 portions/day group, 30 participants (77 %) reported an increase in FV consumption at 18-months, one reported no change (2 %) and eight (21 %) reported a decrease in FV consumption.

**Table 1:** Self-reported FV intake at baseline, end of intervention and 18-months post-intervention according to group allocation^a,b^

	Baseline	End of intervention	Post-intervention	Change at end of intervention	Change at post-intervention
F (portions/d)					
2 portions/d	0.6 (0.5)	1.0 (0.6)	1.3 (1.0)	0.4 (0.2,0.6)	0.8 (0.4,1.1)
5 portions/d	0.7 (0.5)	4.1 (1.2)	2.1 (1.2)	3.4 (3.0,3.7)	1.4 (1.0,1.8)
V (portions/d)					
2 portions/d	0.8 (0.4)	0.8 (0.3)	1.2 (0.6)	0.0 (−0.1,0.1)	0.4 (0.2,0.6)
5 portions/d	0.8 (0.3)	2.0 (0.9)	1.5 (0.5)	1.2 (0.9,1.5)	0.7 (0.6,0.9)
FV (portions/d)					
2 portions/d	1.4 (0.7)	1.8 (0.6)	2.6 (1.5)	0.4 (0.1,0.6)	1.2 (0.7,1.6)
5 portions/d	1.4 (0.5)	6.0 (1.3)	3.6 (1.3)	4.6 (4.2,5.0)	2.1 (1.7,2.6)

^a^F, fruit; V, vegetables; FV, fruit and vegetables; ^b^Values are mean (SD) or mean change (95 % CI). Changes were calculated as end of intervention – baseline and post-intervention – baseline values

Table 2 presents the change in factor scores for barriers to FV consumption between baseline and the end of the intervention and between baseline and 18-months post-intervention. At the end of the intervention, there was an increase in liking of FV (F(1,79) = 5.43, *p* = 0.02) and ease of consumption (F(1,79) = 4.88, *p* = 0.03) with no difference between the 2 portions/day group and 5 portions/day group (F(1,79) = 0.33, *p* = 0.57 and F(1,79) = 0.43, *p* = 0.51, respectively). Awareness of current FV recommendations also increased significantly between baseline and the end of the intervention in the 5 portions/day group compared to the 2 portions/day group (F(1,79) = 4.67, *p* = 0.03). There were no other significant changes evident in barriers to FV consumption between baseline and the end of the intervention.

**Table 2:** Barriers to FV consumption at baseline, end of intervention and 18-months post-intervention^a,b^

	Group allocation at baseline	Baseline	End of intervention	Post-intervention	Change at end of intervention	Change at post-intervention
Liking	2/d	0.82 (0.87)	1.32 (0.74)	1.15 (0.88)	0.51 (0.76)	0.34 (0.77)
	5/d	1.05 (0.82)	1.69 (0.49)	1.54 (0.53)	0.65 (0.65)	0.49 (0.69)
Ease	2/d	1.33 (0.60)	1.55 (0.52)	1.64 (0.49)	0.21 (0.72)	0.31 (0.80)
	5/d	1.34 (0.73)	1.56 (0.49)	1.73 (0.36)	0.21 (0.65)	0.39 (0.70)
Difficulties	2/d	−0.89 (0.78)	−0.75 (0.88)	−1.53 (0.53)	0.15 (0.92)	−0.63 (0.77)
	5/d	−1.01 (0.74)	−1.26 (0.62)	−1.50 (0.54)	−0.24 (0.72)	−0.49 (0.83)
Awareness	2/d	1.74 (0.60)	1.58 (0.86)	1.97 (0.16)	−0.16 (0.92)	0.24 (0.59)
	5/d	1.70 (0.65)	1.98 (0.16)	1.82 (0.68)	0.28 (0.68)	0.13 (0.72)
Willingness to change	2/d	1.47 (0.59)	1.42 (0.61)	1.10 (0.76)	−0.05 (0.69)	−0.37 (0.95)
	5/d	1.41 (0.63)	1.50 (0.56)	1.34 (0.60)	0.09 (0.63)	−0.07 (0.80)

^a^FV, fruit and vegetable; ^b^Values are mean score ± SD

At 18-months post intervention, relative to baseline, there was a greater liking of FV reported (F(1,75) = 8.38, *p* < 0.01), with no difference between groups (F(1,75) = 0.10, *p* = 0.75). Perceived ease in consuming FV increased (F(1,75) = 11.54, *p* = 0.001) while difficulties associated with consuming FV decreased (F(1,75) = 14.35, *p* < 0.001), and again no differences were found between groups (smallest F(1,75) = 0.14, *p* = 0.71). An interaction (F(1,75) = 9.31, *p* < 0.01) also revealed a significant increase in awareness of FV recommendations in the 2 portions/day group at 18-months (t(37) = 2.48, *p* = 0.02) but no change in awareness in the 5 portions/day group (t(39) = 1.09, *p* = 0.28). There were no other significant changes in barriers to FV consumption between baseline and post intervention or 18-months follow-up.

Regression analyses furthermore revealed FV consumption at follow-up, and change in FV consumption from baseline to follow-up to be significantly predicted by change in liking from baseline to follow-up (β = 0.27, *p* = 0.05 and β = 0.28, *p* = 0.05 respectively), change in awareness from baseline to follow-up (β = −0.28, *p* = 0.04, and β = −0.27, *p* = 0.05 respectively) and intervention group (both analyses: β = 0.26, *p* = 0.03). FV consumption at the end of the intervention, and change in FV consumption from baseline to the end of the intervention was predicted only by the intervention group (2 vs. 5 portions/day) (β = 0.90, *p* < 0.01 and β = 0.89, *p* < 0.01 respectively).

## Discussion

The current study examined the effect of participating in a FV intervention study on longer-term FV consumption and on changes in barriers to FV consumption. We showed that participating in a FV intervention resulted in longer-term positive changes in FV consumption. Although there was some attenuation of change in FV consumption at 18-months, mean FV intakes in both groups were still greater than those reported at baseline (mean increase of 1.2 (95 % CI 0.7, 1.6) and 2.1 (1.7, 2.6) portions/day in the 2 portions/day and 5 portions/day group, respectively). At 18-months, FV intakes remained greater in the 5 portions/day group compared to the 2 portions/day group, with the 5 portions/day group consuming on average one more portion of FV/day compared to the 2 portions/day group, therefore the greater effect on FV consumption was in those who had increased their FV intake as part of the study.

Significant decreases in barriers to FV consumption were also observed, both at the end of the intervention and post-intervention. This increase in FV consumption and liking of FV, in both groups, at both timepoints, may reflect participation in the study, the study design and the approaches used in the delivery of the intervention. Participants were not restricted in their choice of FV; rather they were encouraged to try a variety of FV which they may not necessarily have consumed prior to the intervention. As FV were provided free-of-charge throughout the intervention, participants may have been exposed to new or unfamiliar FV, which may have resulted in increased liking and led to sustained increases in FV consumption. Indeed, previous interventions that involved tasting fruit, vegetables or FV dishes and interventions that involved trying different recipes and methods of cooking have similarly been shown to result in improved FV consumption [21–23]. A recent cross-sectional study which examined barriers to increasing FV intakes in older adults also found that greater FV consumption was significantly associated with greater liking for FV [18].

The increased perception in ease in consuming FV at the end of the intervention and after 18-months in both groups, and the reduction in perception of difficulties associated with consuming FV after 18-months may again reflect the approaches used to encourage compliance during the intervention. Apart from receiving a weekly delivery of FV, all participants received practical advice regarding FV storage and preparation, recipes and ideas for incorporating FV into their diet and were encouraged to self-monitor their FV intake during the intervention. This repeated exposure to a variety of FV, coupled with the ongoing support may have resulted in participants establishing a routine so that by the end of the intervention they found FV easier to store, prepare, cook, and consume and it was easier to choose a variety of FV when shopping. This may have resulted in a behaviour change that they were able to maintain in the longer-term. It is also possible that the pragmatic nature of this study and the encouragement to try new FV had a positive effect within the 2 portions/day group, in that by the end of the intervention and at post-intervention they had also increased their FV intake, albeit to a lesser extent than the 5 portions/day group. These findings support those of another recent study conducted in older adults who were low fruit consumers whereby repeated exposure to fruit over a 5-week period resulted in a significant increase in fruit intake and overall FV intake [23].

Participants in the 5 portions/day group became more aware of government recommendations at the end of the intervention. This was a key message for the intervention group and the repeated daily exposure of this message may have translated into greater awareness of the ‘5-a-day’ recommendations. At the end of the intervention, the 2 portions/day group were given the same verbal and written information as the 5 portions/day group received at baseline, regarding the ‘5-a-day’ message which may explain their increased awareness at the post-intervention assessment. The association between change in awareness and FV intake at follow-up in the regression analyses is likely a result of the simultaneous adjustment for all barriers in these analyses, such that, the impact on FV consumption as a result of liking, ease and difficulties were greater in those with lower awareness at baseline. Although willingness to change showed no change after 18-months, this may simply relate to the fact that since participating in the study, participants had increased their FV intake and therefore, they may have felt they were consuming enough FV and were unlikely to make any further changes to their FV consumption. At baseline, regardless of group allocation, only 32 % (*n* = 24) of participants considered themselves to be consuming enough FV compared to 77 % (*n* = 60) and 60 % (*n* = 47) at the end of the intervention and after 18-months, respectively. The results however showed that after 18-months, the majority (85 % (*n* = 68)) of participants were still consuming below the recommended ‘5-a-day’.

Although many studies have successfully demonstrated short-term increases in FV consumption in response to a FV intervention, researchers have rarely gone back to participants to assess whether changes in FV consumption have been maintained a year or more after the intervention ended. Indeed, a recent systematic review which examined maintenance of behaviour change following dietary and physical activity interventions noted that out of 157 trials published since 2000, only 35 % of them included a measure of maintenance of behaviour change [24]. Although a number of studies have shown similar results to ours, in terms of increased FV consumption following participation in a FV intervention [9–13, 25], the majority of these studies had shorter follow-up (3 months–12 months) [9–12] and were conducted in younger age groups [11, 13]. Consistent with our findings, these studies reported that the intervention groups maintained higher intakes of FV post-intervention compared to the control group. They also observed increases in FV intakes in the control group at follow-up compared to baseline [9–13, 25]. In these studies, the magnitude of change in FV intake for both intervention and control groups was similar to those observed in the current study. Such changes in FV intake may have clinical significance [26].

Our findings support those of other FV intervention studies which assessed barriers to FV consumption and showed that participating in a FV intervention was effective in reducing barriers [16, 27, 28]. However, unlike our study which examined both the short- (16-weeks) and longer-term (18-months) changes in a range of barriers to FV consumption in older adults, most of the previous studies were conducted in younger population groups, did not examine longer-term changes in barriers and tended to focus on single or fewer barriers to FV consumption. However, it must be remembered that, unlike previous studies, the participants in our study were selected to have low FV consumption, therefore the barriers observed may have differed from those in populations already consuming larger amounts of FV.

A strength of our study was the high retention rate throughout the intervention and post-intervention period. This may reflect the approach used in the intervention where compliance was encouraged by attempting to address and overcome several perceived barriers to FV consumption whilst allowing participants to continue with their usual lifestyle. This may have contributed to the dietary change being maintained, albeit to a lesser degree, after discontinuation of the intervention. The high retention rate may also reflect the age of the participants in that they possibly had more time available to take part in the study. A further strength is that maintenance of change was assessed following an 18-month period during which no contact was made with participants.

A study limitation was the reliance on self-reported dietary intake. While the diet history is a well-validated, dietary assessment method for assessing habitual dietary intake, it is prone to measurement error which may have resulted in under- or, more likely, over-reporting of FV intake. Unlike the intervention period, where micronutrient status was measured alongside dietary intake at the start and end of the intervention, the 18-month post-intervention assessment relied solely on self-reported dietary intake for pragmatic reasons. We also cannot rule out the possibility that participants over-reported their FV intake at the 18-month assessment given that they had previously participated in the intervention. It is also possible that the recruitment of healthy, free-living participants and the approaches used to recruit participants onto the study may have introduced potential recruitment bias. There are also limitations with the barriers to FV consumption questionnaire. While the current study assessed numerous barriers to FV consumption, this data was based on responses to closed response questions which were derived from previous papers investigating barriers to FV consumption in other populations. The reliability of the barriers questionnaire for capturing all possible reasons for not increasing FV consumption is therefore questionable [3]. It is also important to acknowledge that other factors such as socio-economic status and education may also impact on FV intake and on barriers to FV intake and would be worth exploring in future work. Finally, our research was conducted in an older population who were low FV consumers and therefore our results may not be generalizable to other population groups.

## Conclusions

The findings of this study offer encouraging evidence regarding the efficacy of FV interventions in encouraging behaviour change in FV consumption. We have shown that regardless of group allocation, participating in a FV intervention which offers individual support, reinforces the ‘5-a-day’ message and provides practical solutions to perceived barriers is effective in increasing FV consumption, which can be sustained for at least 18-months after cessation of the intervention, and can help reduce perceived barriers to FV consumption. This approach may enable participants to take control of their own dietary intake, thereby facilitating a behaviour change that can be sustained over time. While further intervention studies with longer-term follow-up assessments are required to fully determine the sustained effects of participation, our findings demonstrate the potential value of practical support including repeated exposure to different types of FV and individual encouragement as a means of overcoming barriers to increased FV consumption. These findings may help guide future interventions aimed at changing dietary behaviour and should be considered when designing strategies to increase FV consumption at a population level.

## Abbreviations

95 % CI: 95 % Confidence interval
ADIT: Ageing and Dietary Intervention Trial
BMI: body mass index
d: day
FV: fruit and vegetables
mo: month
SD: standard deviation
wk: week
y: year
